# Mesenchymal Stromal Cell-Based Therapies in Sepsis-Induced Acute Lung and Kidney Injury: Current Advances and Perspectives

**DOI:** 10.3390/ijms27156990

**Published:** 2026-08-04

**Authors:** Carla M. da Silva, Mayck M. A. da Silva, Marcelo M. Morales

**Affiliations:** 1 Laboratory of Cellular and Molecular Physiology, Carlos Chagas Filho Institute of Biophysics, Federal University of Rio de Janeiro, Rio de Janeiro 21941-902, RJ, Brazil; medeiroscarla@biof.ufrj.br; 2 Laboratory of Pulmonary Investigation, Carlos Chagas Filho Institute of Biophysics, Federal University of Rio de Janeiro, Rio de Janeiro 21941-902, RJ, Brazil; medeirosmayck@biof.ufrj.br

**Keywords:** sepsis, mesenchymal stromal cell, cell therapy

## Abstract

Sepsis is a life-threatening syndrome characterized by severe immune dysregulation, frequently culminating in acute respiratory distress syndrome (ARDS) and acute kidney injury (AKI). Current supportive therapies fail to reverse the underlying pathophysiological damage. However, mesenchymal stromal cells (MSCs) have emerged as a promising therapeutic frontier due to their robust immunomodulatory, anti-inflammatory, and tissue-regenerative properties. Despite compelling preclinical evidence, translating these benefits into consistent clinical efficacy remains a major challenge. This review critically examines the biological and anatomical barriers limiting the efficacy of MSCs, particularly the pulmonary first-pass effect, which restricts the systemic delivery of viable cells to distant organs such as the kidneys. To overcome these physical limitations, we highlight the recent paradigm shift toward nanoscale, cell-free therapies, specifically MSC-derived extracellular vesicles (MSC-EVs). EVs effectively bypass pulmonary sequestration and thromboembolic risks, exerting their potent therapeutic effects through the horizontal transfer of bioactive cargo, notably microRNAs, to reprogram cellular fate and restore immune homeostasis. We also discuss the critical need for rigorous clinical trial designs, scalable good manufacturing practice protocols, and the integration of a precision medicine approach. Ultimately, incorporating validated biomarkers for targeted patient stratification will be the decisive step in unlocking the full therapeutic potential of MSCs and their derivatives in critical care.

## 1. Sepsis Definition and Epidemiology

The term sepsis originated from the Greek sêpsis (σ’ηψις), which means putrefaction. It was described by Hippocrates as a dangerous and odorous biological impairment that could occur in an organism [1]. For many years, the plurality of definitions used to characterize a patient with sepsis constituted a significant limitation to its better understanding. The nomenclature used previously, such as septicemia, septic syndrome, or generalized infection, did not provide clarity from both a care and a scientific point of view [2]. In this context, the international scientific community has refined the terminology and diagnostic criteria for sepsis to try to establish consensus. This standardization has evolved through three main revisions [2,3,4]. The most recent definition of sepsis is life-threatening acute organ dysfunction due to infection [2].

Despite extensive research, sepsis remains a critical global health crisis. Clinical studies based on the Sepsis-3 definition report an individual mortality rate of approximately 25% [2,5], but the absolute rate on a global scale is even more alarming. According to the latest Global Burden of Disease estimates, sepsis affects approximately 166 million individuals annually and is responsible for 21.4 million deaths, which is nearly 31.5% of all global mortality [6,7].

Beyond its clinical toll, sepsis imposes a profound economic burden. Systematic reviews indicate that hospital-related costs vary significantly by region, ranging from €1101 to over €91,000 per patient [8]. In high-income settings, the average cost per case exceeds US$32,000, and hospital stays typically last twice as long as those for other fatal conditions [9]. Furthermore, survivors often face post-intensive care syndrome, which is characterized by a higher risk of premature death and severely reduced quality of life [10,11,12]. Collectively, these data position sepsis as one of the most clinically and financially burdensome conditions in modern medicine.

There are three fundamental challenges in the diagnosis of sepsis: (1) the lack of specificity of clinical signs, which frequently mimic other pathological conditions; (2) the lack of biomarkers with ideal sensitivity and specificity, given the pathophysiological complexity of the syndrome; and (3) the marked heterogeneity of the disease, with no uniform cause, phenotype, or biological signatures [13,14]. These obstacles demand urgent attention because early detection and immediate therapeutic intervention, ideally within the first 3 h, are crucial for optimizing clinical outcomes and improving the prognosis [14,15,16,17].

## 2. Pathogenesis of Sepsis and Organ Involvement

The pathophysiology of sepsis is characterized by its complexity, occurring when the pathogen manages to evade the host’s defense mechanisms and continues to stimulate and damage the body’s cells. This process results in a dysfunction of the immunological mechanisms initially activated to promote protection, which becomes detrimental due to the failure to restore homeostasis. This scenario leads to persistent hyperinflammation, accompanied by a state of immunosuppression and failure to restore immune balance [18,19].

The inflammatory disorder triggered by sepsis, associated with vascular hypercoagulability, increased endothelial permeability, and peripheral vasodilation, can affect the function of multiple organs. This process causes various clinical signs and symptoms in the patient, such as fever, increased respiratory and/or heart rate, hypotension, and mental confusion [2]. Among the various organs affected during a severe septic episode, the lungs are affected in approximately 40% of patients, and the kidneys in 50% [20]. Lung and kidney involvement, either by infectious agents present in the bloodstream or by the resulting systemic inflammation, leads to the development of pathological conditions known as acute respiratory distress syndrome (ARDS) and acute kidney injury (AKI). ARDS and AKI are serious conditions that affect many patients with sepsis, overburdening intensive care units; in addition, these conditions are associated with higher mortality rates [21,22,23].

### 2.1. Acute Respiratory Distress Syndrome

ARDS is a life-threatening complication of sepsis, characterized by severe hypoxemia and mortality rates often exceeding 40% [24,25]. The biological hallmark of the syndrome is the severe inflammatory disruption of the alveolar–capillary interface, which results in microvascular hyperpermeability, impaired fluid clearance, and protein-rich pulmonary edema [14,26].

Critically, ARDS is a highly heterogeneous syndrome. Beyond the classic anatomical distinction between direct (pulmonary) and indirect (extrapulmonary) insults, recent advances have identified distinct molecular subphenotypes. Notably, the hyperinflammatory phenotype, marked by increased interleukin (IL)-6 and soluble tumor necrosis factor (TNF) receptor-1, is associated with significantly higher mortality and divergent therapeutic responses compared with the hypoinflammatory phenotype [27,28,29]. This heterogeneity imposes a narrow and dynamic therapeutic window. Early interventions must dampen the initial hyperinflammatory surge and stabilize the endothelial–epithelial barrier. However, as the disease rapidly progresses toward immune paralysis or fibroproliferation, broad immunosuppression can become detrimental [30].

Therefore, effective therapies must transcend simple anti-inflammatory actions to actively promote tissue resolution, such as accelerating epithelial repair and repolarizing alveolar macrophages toward an M2-like profile, mechanisms for which mesenchymal stromal cells (MSCs) and their extracellular vesicles (EVs) show substantial promise [24,31]. Furthermore, because ARDS frequently acts as a catalyst for multiple organ dysfunction syndrome (MODS), these systemic therapies must also address distant organ crosstalk, particularly mitigating concurrent AKI [32].

### 2.2. Acute Kidney Injury

Sepsis-associated acute kidney injury (AKI-S) is a devastating complication that increases hospital mortality up to eightfold and triples the risk of subsequent chronic kidney disease [33,34]. Often manifesting as an early clinical sign of septic shock, sepsis accounts for up to 70% of all cases of AKI in critically ill patients [35,36].

Rather than pure ischemic necrosis, the pathogenesis of AKI-S is driven primarily by a highly heterogeneous synergy between systemic hyperinflammation and severe microvascular dysfunction. During the early inflammatory phase, the activation of Toll-like receptors (TLRs) on renal tubular epithelial cells (TECs) triggers robust nuclear factor (NF)-κB-mediated cytokine release, oxidative stress, and peritubular leukocyte recruitment [37,38]. Faced with this intense biological alarm, TECs undergo a survival-driven metabolic reprogramming, often characterized as a functional “shutdown” [39,40]. This bioenergetic arrest, marked by mitochondrial dysfunction and vacuolization, halts renal excretory function but preserves overall cell viability.

Consequently, the successful management of AKI-S relies on a critical therapeutic window and an understanding of distinct inflammatory phenotypes. Effective therapies must reach the renal microcirculation during the acute hyperinflammatory phase to blunt the cytokine storm while simultaneously rescuing the mitochondrial bioenergetics of “shutdown” TECs. Intact MSCs possess this dual immunomodulatory and metabolic capability, but their therapeutic application in AKI is severely constrained by the pulmonary first-pass effect, which entraps the large cells before they can engraft in the kidneys. In this context, nanoscale EVs have emerged as the ideal therapeutic platform. Capable of freely navigating to the inflamed renal parenchyma, source-specific EVs can deliver targeted epigenetic cargo to silence TLR networks and transfer functional organelles to reverse TEC metabolic exhaustion, offering a precision-guided resolution to AKI.

## 3. Therapies Used in Sepsis

Sepsis is a major global health concern and is one of the most feared complications for hospitalized patients; it is the direct or indirect cause of about 50% of all hospital deaths. As a time-dependent disease, sepsis requires rapid recognition and a standardized approach for optimal treatment [41].

The current management of sepsis is based primarily on eradicating the invading microorganism through prompt administration of antibiotics and achieving adequate control of the source. In addition, management of the hemodynamic, ventilatory, and metabolic changes resulting from systemic inflammation is also included. Even so, strategies aimed at reducing tissue damage remain limited, focusing on the early use of vasoactive amines, protective mechanical ventilation, prone positioning, and alveolar recruitment maneuvers, as well as extracorporeal circulation support in refractory situations [5].

However, these therapeutic approaches used to minimize the effects generated by sepsis have not yet been entirely successful in blocking or reversing the exacerbation of the inflammatory response and subsequent loss of vital tissue function efficiently, representing a major challenge in controlling the progression of sepsis. It is also believed that immunosuppression is the predominant driving force in morbidity and mortality in sepsis [42].

Therefore, it is considered that a promising therapy for the treatment of sepsis must act by mitigating the tissue damage that occurs in the early pro-inflammatory phase and, simultaneously, modulating the immune system to neutralize the imbalance characterized by the late predominance of immunosuppression [42,43].

In this context, the immune reprogramming properties of cell therapy using MSCs are an emerging therapeutic strategy for sepsis and associated organ dysfunction.

### 3.1. Cellular Therapy

MSCs are multipotent and immune-evasive cells that exhibit low immunogenicity and do not cause allogeneic rejection, a significant advantage over autologous administration, which is often limited by the time required for culture and expansion [31,44]. Defined by the International Society for Cell Therapy, their fundamental characteristics include adhesion to plastic, fusiform morphology, and the potential for trilineal differentiation into chondrocytes, adipocytes, and osteoblasts in vitro. In addition, they must present a specific surface signature, with positive expression of markers such as CD29, CD44, CD73, CD90, and CD105, and the absence of hematopoietic markers such as CD45, CD34, CD14 or CD11b, CD79α or CD19, and HLA-DR, in strict accordance with the modern consensus criteria established by the International Society for Cell & Gene Therapy (ISCT), which acknowledges that these core markers are expressed regardless of the tissue source, despite functional and differentiation differences among them [45,46].

MSCs are known to be heterogeneous [47] and can be isolated from multiple tissues, including bone marrow (BM), adipose tissue (AT), umbilical cord (UC), placenta, lung tissue, and menstrual blood [48,49]. Studies have revealed that variations in gene expression, genomic stability, secretome, and cell surface proteins depend on the isolation source, which can directly influence their immunomodulatory properties [50,51,52,53]. Despite these biological and manufacturing heterogeneities, MSCs continue to dominate the landscape of translational cell therapy, with more than 1900 registered trials in the ClinicalTrials.gov database (accessed 27 March 2026).

The therapeutic potential of MSCs is based on their broad physiological effects, ranging from the maintenance of tissue homeostasis to complex regeneration processes [54,55,56], and is intrinsically associated with their ability to migrate to areas of injured tissue and promote the secretion of factors with immunomodulatory, antimicrobial, anti-apoptotic, and trophic properties [52,57]. These immunomodulatory activities make MSCs suitable for various therapeutic applications, because the integration of these biological mechanisms directly contributes to tissue regeneration and modulation of the inflammatory microenvironment [58].

MSC-mediated immunomodulation is driven by a sophisticated synergy between direct cell-to-cell contact and the secretion of bioactive soluble factors [59,60]. MSCs reveal their immunomodulatory potential through functional alterations in monocytes/macrophages, dendritic cells, T cells, B cells, and natural killer cells [57,61,62,63,64]. Furthermore, studies have demonstrated that MSCs administered as therapy act through paracrine/endocrine mediators, generating protective effects in cells and tissues [65].

The mechanism by which MSCs migrate to injured regions is not yet fully elucidated, but it appears to depend on the passage of these cells through capillaries, where their speed is reduced. This process allows interaction with cytokines, activation of integrins, cell adhesion, tracking, and targeting to the site of injury [66,67].

Although initial studies focused on the direct role of MSCs in cell regeneration, the current understanding is that their administration exerts tissue-protective effects predominantly through paracrine mediators that act on target cells [68]. This phenomenon is believed to occur through mechanisms such as the release of soluble factors, extracellular vesicles, and organelle transfer, such as mitochondria [69], generating high therapeutic expectations in areas such as sepsis, transplant medicine, and autoimmune diseases, with encouraging preclinical results [64,67,70,71,72,73,74].

In addition to these properties, the ability to regulate the immune system is a fundamental characteristic of MSCs. Although cell–cell contact may be relevant, the literature corroborates the predominance of secretion of paracrine factor in these immunoregulatory mechanisms [75]. The involvement of these soluble factors has been demonstrated in experiments [76] with semipermeable membranes (Transwell), which showed immune modulation even in the absence of physical contact between cells. Various mediators, including TGF-β1 [77,78], IL-6, IL-10 [76], hepatocyte growth factor [77,79], prostaglandin E2 [80], and indoleamine 2,3-dioxygenase [81,82], are implicated in the immunomodulation of T and B cells [75,83,84,85,86], in cell maturation of dendritic cells [87], and natural killer cell proliferation [88,89,90]. This secretome modulates the inflammatory response by suppressing exacerbated immune activity and promoting the proliferation of endothelial cells and fibroblasts, essential for tissue repair and extracellular matrix remodeling [75].

Extensive preclinical evidence has demonstrated that MSCs significantly reduce lung injury and improve survival in various animal models of ARDS through multiple mechanisms, including anti-inflammatory, anti-apoptotic actions, epithelial and endothelial repair, increased bacterial clearance, and antioxidant activity [91,92,93,94,95,96,97,98,99,100,101,102]. These encouraging preclinical results have driven clinical translation, historically establishing BM-derived MSCs as the most widely used source in recent decades; however, UC- and AT-derived MSCs have been increasingly explored. According to ClinicalTrials.gov registries, these three sources of MSCs are the most tested in various clinical settings [47], consistently demonstrating significant therapeutic benefits. In this scenario, the application of these strategies demonstrates crucial relevance to test MSCs as a treatment for ARDS. The effectiveness of these cells in modulating the pulmonary inflammatory response and improving clinical outcomes in patients is highlighted in Table 1 [103,104].

Despite encouraging results in experimental models, the clinical application of MSCs faces critical gaps regarding dose definition, route of administration, tissue source, timing of intervention, and treatment duration. In this scenario, safety concerns persist related to the administration of high cell doses, which may lead to risks of pulmonary embolism [124,125] or ischemic events; a Phase IIa study on patients with pulmonary fibrosis who received high doses of BM-MSCs (8 doses of 2 × 10^8^ cells, totaling 1.6 × 10^9^ cells per patient) reported one case of ischemic stroke after MSC infusion [126]. Although the administration of BM-MSCs has generally proven safe, evidence of efficacy in respiratory or kidney diseases and sepsis remains limited [111,127], indicating significant barriers to clinical translation [128]. This limited therapeutic potential is largely attributed to suboptimal dosing [111], delayed administration during the clinical course of the disease [129,130], poor in vivo cell survival rate [131], and compromised intrinsic biological activity of the infused cells [132].

A critical barrier in the clinical translation of MSCs lies in their physical dimensions and the consequent anatomical biodistribution after systemic administration. With a cell diameter ranging from 15 to 50 µm, intravenously infused MSCs are rapidly and predominantly sequestered within the pulmonary capillary network, a phenomenon known as the pulmonary first-pass effect.

In the context of ARDS, this mechanical entrapment serves as an advantageous, naturally targeted delivery system. By accumulating directly at the site of the injured alveolar–capillary barrier, MSCs can locally exert their robust paracrine, immunomodulatory, and endothelial-stabilizing effects. This anatomical alignment between the site of cell sequestration and the primary organ injury provides a strong biological rationale for the safety and early physiological improvements observed in pulmonary-focused trials [108].

Conversely, this obligatory pulmonary clearance severely restricts the systemic delivery of viable cells to distant organs, presenting an impaired translational barrier for treating conditions such as AKI. Attempts to bypass the pulmonary circulation to achieve higher renal engraftment have highlighted significant safety and efficacy concerns. Notably, the Phase II trial by Swaminathan et al. [106] used an intra-aortic delivery route to administer MSCs in patients with AKI post-cardiac surgery. However, this strategy failed to improve the time to renal recovery and paradoxically demonstrated a non-significant trend toward worse clinical outcomes, including higher mortality and increased need for dialysis.

From a pathophysiological perspective, this clinical failure underscores a critical risk: forcing large cellular products into an already inflamed, edematous, and ischemic renal microcirculation may precipitate microvascular occlusion and microthrombosis, thereby exacerbating tissue hypoxia rather than resolving it. Overcoming the physical and biological limitations imposed by the first-pass effect remains imperative. Faced with such challenges, new strategies are emerging to optimize therapeutic efficacy through two main translational avenues. The first is a paradigm shift toward nanoscale, cell-free therapies (such as EVs), which can freely navigate the systemic microvasculature without the thromboembolic risks associated with intact MSCs.

### 3.2. Translational Challenges

Despite the robust immunomodulatory and regenerative properties demonstrated by MSCs in animal models, translating these benefits into clinical efficacy has proven exceedingly challenging. A critical examination of recent clinical trials (Table 1) reveals a stark contrast between preclinical promise and clinical reality, characterized predominantly by neutral or negative efficacy outcomes.

In the context of ARDS, although Phase I dose-escalation trials confirmed the overall safety of intravenous administration of MSCs [111], subsequent adequately powered Phase II trials failed to meet primary efficacy endpoints. Most notably, the Phase IIa START trial [112], evaluated a single dose of 10 million BM-MSCs/kg in 60 patients with moderate to severe ARDS. Although safe, the intervention did not yield statistically significant improvements in 60-day mortality or ventilator-free days compared with placebo. Similarly, the MUST-ARDS trial [107] utilizing multipotent adult progenitor cells demonstrated safety but failed to significantly improve day-28 mortality or ventilation parameters. In the realm of AKI, results have been equally frustrating. In a Phase II trial [106], which used an intra-aortic delivery of MSCs after cardiac surgery, not only was there no improvement in the time to renal recovery, but there was also a non-significant trend toward worse clinical outcomes.

This recurrent translational failure across different organ dysfunctions can be attributed to several critical limitations in current trial designs. First, the extreme clinical and molecular heterogeneity of sepsis and ARDS is rarely accounted for. Preclinical models typically use genetically identical animals with synchronized disease onset, whereas human trials enroll highly heterogeneous cohorts. Administering broad immunosuppressive cells to a patient who has already entered the immune paralysis phase of sepsis may be ineffective or even detrimental. Second, the choice of endpoints plays a confounding role. Most trials rely on hard clinical endpoints (e.g., 28-day or 60-day all-cause mortality), which are heavily influenced by preexisting comorbidities and intensive care complications, thereby diluting the specific biological effect of the cells.

Third, manufacturing and biological viability remain severely overlooked. Many negative trials, including the START trial, utilized freshly thawed cryopreserved cells administered directly at the bedside. Emerging evidence indicates that MSCs undergo a transient functional impairment post-thawing, characterized by impaired immunomodulatory secretome, reduced survival, and high susceptibility to complement-mediated lysis in the bloodstream, rendering them biologically inert precisely during the critical early window of hyperinflammation [133].

Therefore, recognizing these clinical failures is essential. Advancing the field requires shifting from a generic one-size-fits-all cell infusion protocol toward precision-guided therapies. This involves standardizing post-thaw recovery protocols, exploring multiple dosing regimens, and utilizing transcriptomic or protein biomarkers to stratify patients, ensuring cells are infused only during the hyperinflammatory phenotype where their immunomodulatory action is most required.

Although this review specifically addresses sepsis-induced organ injury, it is important to recognize that the COVID-19 pandemic represented a unique opportunity for the clinical evaluation of MSC-based therapies in patients with severe systemic inflammation and acute lung injury. As a result, some of the largest and most informative clinical trials investigating MSC therapy were conducted in COVID-19-associated ARDS. While these studies do not directly address sepsis, they substantially expanded current knowledge regarding the safety, feasibility, biological activity, and potential therapeutic effects of MSCs in uncontrolled inflammatory syndromes. Therefore, they have been incorporated into this review as relevant translational evidence, while acknowledging that direct extrapolation to sepsis should be made cautiously because of important differences in disease pathophysiology (Table 1) [94,115,116,117,121].

### 3.3. Extracellular Vesicles

EVs are nanoscale lipid-bilayer particles and lack a functional nucleus [134]. They are emerging as a promising cell-free therapeutic platform for sepsis. Ranging from 10 to 1000 nm in diameter, EVs effectively bypass first-pass pulmonary sequestration and the thromboembolic risks associated with intact MSCs [134,135]. Translationally, EVs offer biological stability under cryopreservation, low immunogenicity (minimal expression of major histocompatibility complex), and an absence of tumorigenic risk. Their structural robustness protects bioactive cargoes (mRNA, miRNAs, proteins, and organelles) from the hyperinflammatory systemic milieu, ensuring effective biodistribution to distant organs [136]. Table 2 summarises those MSC and MSC-derived EVs characteristics.

To systematically understand the therapeutic effects of MSC-EVs upon systemic administration in sepsis, their mechanisms of action can be categorized by some of specific bioactive factors they deliver to target cells:

- Organelle Transfer and Bioenergetic Rescue: A central pillar of EV therapy is the horizontal transfer of functional mitochondria or mitochondrial fragments to injured cells [137]. This mechanism is a determinant for survival in MODS, where ATP preservation is critical [138,139,140]. In the lung, EVs restore oxidative phosphorylation and alveolar–capillary barrier integrity, mitigating edema and inflammation [141,142]. Simultaneously, in AKI, this mitochondrial delivery rescues tubular metabolism, directly combating sepsis-induced bioenergetic collapse [137].
- Protein-Mediated Immune Repolarization: Immunologically, EVs transfer specific proteins that orchestrate macrophage repolarization from a pro-inflammatory state toward a pro-resolving M2 phenotype. This shift fundamentally alters the systemic inflammatory milieu by increasing the secretion of IL-10 while significantly reducing pro-inflammatory cytokines such as TNF-α and IL-8 [143,144].
- Epigenetic Regulation via microRNAs: The ‘antiseptic’ modulation provided by EVs is potently driven by the transfer of specific miRNAs that reprogram cellular fate. For instance, in sepsis-induced AKI, EV-derived miR-146b inhibits the IRAK1/NF-κB inflammatory pathway [145], and miR-342-5p suppresses the TLR9 receptor, accelerating protective autophagy [146]. Furthermore, the targeted delivery of miR-223 dismantles the NLRP3 inflammasome, effectively halting pyroptosis in both the pulmonary endothelium and renal parenchyma [147].

Collectively, the synergistic action of these factors allows EVs to effectively blunt the cytokine storm, restore metabolic homeostasis, and promote tissue repair in distant organs.

The therapeutic profile of EVs is fundamentally dictated by their tissue of origin. Although MSC-EVs broadly share regenerative capabilities, their specific biological functions and disease-targeting efficiencies vary significantly depending on the source tissue [148]. This functional divergence is intrinsically linked to their distinct molecular cargoes, as demonstrated by recent proteomic analyses. Adipose-derived EVs are uniquely enriched in proteins that regulate the immune system and promote angiogenesis, making them highly attractive for mitigating hyperinflammation. UC-derived EVs exhibit a proteomic signature predominantly associated with tissue damage repair and wound healing, which is critical for restoring epithelial and endothelial barriers in ARDS and AKI. Conversely, BM-derived EVs are mainly enriched in proteins involved in cell communication and developmental growth [149].

Recognizing this intrinsic biological heterogeneity, the field is rapidly advancing toward the development of “designer EVs” through bioengineering. Given that native EVs may carry subtherapeutic concentrations of specific miRNAs, researchers are using strategies such as electroporation, sonication, or cellular preconditioning (priming) to artificially enrich EVs with targeted therapeutic cargoes. Furthermore, surface engineering, such as conjugating targeting peptides or antibodies to the EV membrane, enables precise organotropic delivery [150]. This bioengineered platform promises to overcome the biodistribution limitations of natural EVs, creating highly specific, targeted nanotherapeutics for precision critical care.

A limited number of clinical trials are currently registered on ClinicalTrials.gov investigating EVs for the treatment of ARDS, with a notable absence of studies targeting AKI. Recently, a landmark prospective, multicenter, double-blind, randomized, placebo-controlled Phase II trial evaluated ExoFlo, an allogeneic BM MSC-derived EV product, in patients with COVID-19-associated moderate to severe ARDS. The therapy demonstrated an excellent safety profile with zero treatment-related adverse events. The overall intention-to-treat analysis suggested a potential survival benefit, and statistically significant improvements in 60-day mortality (*p* = 0.034) and ventilator-free days were specifically identified in post hoc subgroup analyses among patients aged 18 to 65 years receiving a 15 mL dose [151]. These findings highlight the therapeutic promise of cell-free modalities and underscore the need for adequately powered Phase III trials to validate these subgroup efficacy signals.

## 4. Discussion and Future Directions

MSCs and their cell-free derivatives have demonstrated solid immunomodulatory, anti-inflammatory, and tissue-regenerative properties in preclinical models of sepsis. However, as highlighted in Table 1, these robust preclinical benefits have not yet fully translated into consistent efficacy in large-scale clinical trials. Notwithstanding, when comparing these two modalities, a critical trade-off emerges between biological adaptability and logistical safety. As outlined in Table 2, the primary advantage of intact MSCs lies in their capacity as ‘living drugs’; they can sense the inflammatory microenvironment and dynamically adapt their immunomodulatory secretome. However, this active biological nature comes with significant disadvantages, including vulnerability to cryopreservation injury, risk of microvascular occlusion (the pulmonary first-pass effect), and complex expansion bottlenecks. Conversely, MSC-derived EVs offer a compelling solution to these physical limitations. Their nanoscale size provides a major advantage by allowing unrestricted systemic biodistribution and targeted delivery to distant organs like the kidneys, without the thromboembolic risks associated with cells. Furthermore, EVs are highly stable, non-replicating, and easier to store. Their main disadvantage, however, is that they are static entities; once administered, EVs cannot continuously adapt to the patient’s fluctuating immune status, relying entirely on the pre-packaged cargo they contain at the time of isolation.

Therefore, the choice between MSCs and EVs in future therapies may depend heavily on the specific phase and phenotype of sepsis being treated. To bridge this translational gap, future trials must be meticulously designed to ensure true clinical applicability. Methodologically, it is imperative to implement rigorous, double-blind, randomized, and placebo-controlled designs to effectively mitigate performance bias. Study protocols must ensure scientific rigor by strictly defining the target population and using robust power analyses to detect clinically meaningful differences. From a manufacturing perspective, therapeutic products must be generated under strict good manufacturing practice (GMP) standards to guarantee batch-to-batch reproducibility and preserve biological potency. In this context, despite the increasing enthusiasm for cell-free modalities, intact MSCs remain a highly active area of clinical investigation. While early clinical translation relied heavily on BM-MSCs, there is now a wealth of data supporting the efficacy of alternative tissue sources. A contemporary analysis of the ClinicalTrials.gov registry reveals a strategic shift toward MSCs derived from alternative tissue sources, including umbilical cord, adipose tissue, and menstrual blood. In addition, placenta-derived decidual stromal cells (DSCs), a specialized stromal cell population with marked immunomodulatory properties, have also been investigated as a related cellular therapy for severe inflammatory lung injury. These alternative sources often provide superior scalability, robust proliferative capacity, and optimized GMP-compliant manufacturing profiles. Recent clinical evidence strongly supports this transition; for instance, trials utilizing UC-MSCs, adipose-derived MSCs, and menstrual blood-derived MSCs have demonstrated significant survival benefits and improved clinical outcomes in patients with severe ARDS, including COVID-19-induced phenotypes. Notably, DSCs have also emerged as a promising, highly immunosuppressive alternative. While initial pilot and propensity score-matched studies have demonstrated clinical potential, such as improved survival in patients with severe COVID-19 ARDS [119,120], large-scale randomized controlled trials (RCTs) are still required to definitively establish their efficacy and safety profile in critical care.

Furthermore, therapeutic success will critically depend on optimizing the window of intervention and the delivery route, strategically accounting for anatomical barriers such as the pulmonary first-pass effect. The translational momentum to bypass these physical limitations is evident in the emerging landscape of cell-free therapies. However, it is imperative to approach the therapeutic superiority of MSC-EVs with caution. While EVs present a compelling physiological rationale in preclinical models, there is currently a lack of concrete data from large-scale, randomized prospective clinical trials supporting their efficacy over intact cellular therapy. A search of the registry reveals several active trials investigating MSC-EVs for ARDS, including large-scale Phase III trials, such as the EXTINGUISH ARDS study (NCT05354141). Until these rigorously designed cohorts provide definitive readouts, conclusions regarding the clinical integration of nanotherapeutics must remain humble, and intact MSC therapies remain the primary benchmark in the field.

Ultimately, while both cell-based and cell-free therapies are advancing in parallel, their definitive success is currently hindered by critical translational barriers. For MSC therapies, the main limitations remain the inconsistencies in cell viability post-thawing (the ‘cryo-stun’ effect), the lack of standardized Good Manufacturing Practice (GMP) protocols across different tissue sources, and the anatomical entrapment of cells in the pulmonary vasculature. To overcome these limitations and address the severe pathophysiological heterogeneity of sepsis, ARDS, and AKI, future trials must undergo a fundamental paradigm shift. The transition from a generic, one-size-fits-all cell infusion protocol to individualized precision medicine depends on robust molecular phenotyping. Recently, longitudinal multi-omics analyses, integrating whole-blood transcriptomics and plasma metabolomics, have provided groundbreaking insights into this biological variance. These studies have successfully identified distinct inflammatory phenotypes (e.g., hyperinflammatory and hypoinflammatory) in critically ill patients with ARDS and sepsis [152]. Furthermore, they reveal that each phenotype is driven by unique molecular mechanisms, such as enhanced innate immune activation coupled with increased glycolysis, or immune and hepatic dysfunction paired with impaired fatty acid oxidation. Recognizing these specific multi-omic signatures and distinct pathways associated with mortality is crucial for targeted interventions. By incorporating such validated biomarkers to stratify patients, researchers can ensure that advanced therapies such as MSCs and EVs are administered specifically to individuals exhibiting the optimal inflammatory and metabolic profile, rather than during immune paralysis, thereby unlocking their full therapeutic potential in critical care.

## Figures and Tables

**Table 1:** Clinical Trials of MSCs in ARDS, AKI, and Sepsis (2014–2026).

Disease	Stem Cell Type	Cell Delivery/ Dose	Time of Delivery	Study Description	Safety Results	Efficacy Results	Reference
AKI (after cardiac surgery)	BM-MSCs	IV/Intra-aortic (low dose?)	During surgery	Phase I, open-label (post-cardiac surgery)	Safe and feasible	*n* = 5 Prevented early deterioration of renal function	[105]
AKI (after cardiac surgery)	BM-MSCs (AC607)	Intra-aortic, single; 2 M cells/kg	48 h after removal from CPB	Phase II, RCT (post-cardiac surgery)	Safe, no long-term AEs	*n* = 135 analyzed No significant improvement in renal recovery, need for dialysis, or mortality; non-significant trend toward worse outcomes in MSC group	[106]
ARDS	MAPCs (Invimestrocel)	IV, single; C1, 300 M; C2 and C3, 900 M	Within 96 h of ARDS diagnosis	Phase I/IIa, dose escalation to RCT	Safe, no major AR	No significant improvement in primary endpoint (day-28 mortality, ventilator-free days)	[107]
ARDS (pneumonia)	MAPCs (Invimestrocel)	IV, single; 900 M cells	Within 72 h of diagnosis	Phase II (ONE-BRIDGE), open-label, RCT (2:1)	Safe, no allergic or serious ARs linked to cells	*n* = 30 Higher VFDs (20 vs. 11) but not statistically significant; trend toward improved survival (day-180 mortality 26% vs. 43%)	[108]
ARDS	Adipose MSCs	IV, single; 1 M cells/kg	Within 48 h of diagnosis	Phase I, RCT	Safe, no MSC- related AEs	Reduced SP-D; no improvement in PaO_2_/FiO_2_, hospital indices, or IL-6/IL-8	[109]
ARDS	BM-MSCs	IV, 6 doses; 2 M cells/kg	Rescue therapy after nonresponse in ECMO support	Compassionate use (*n* = 2)	Safe, no immune reactions	Resolution of respiratory failure; decreased inflammatory markers	[110]
ARDS	BM-MSCs	IV, single; 1.5, or 10 M cells/kg	Within 96 h of diagnosis	Phase I, open-label dose escalation	Well-tolerated	*n* = 9 22% mortality (lower than predicted for severity)	[111]
ARDS	BM-MSCs	IV, single; 10 M cells/kg	Within 96 h of diagnosis	Phase IIa, RCT	Safe, mild side effects	*n* = 60 No statistical difference in mortality or ventilator-free days vs. placebo	[112]
ARDS	CD362+ UC-MSCs (ORBCEL C)	IV, single; up to 400 M cells	Within 48 h of the onset of moderate to severe ARDS	Phase I/IIa, RCT	Safe, no severe MSC-related AEs	No significant differences in oxygenation index or mortality	[113]
ARDS (H7N9 viral pneumonia)	MB-MSCs	IV, multiple (3–4 doses); 1 M cells/kg	Early or late stage of H7N9 infection	Phase I, open-label	Safe, no acute toxicities; long-term safety confirmed	*n* = 61 (17 MSC, 44 control) Significantly lower mortality in MSC group (17.6% vs. 54.5%)	[114]
COVID-19 ARDS	MB-MSCs	IV, 3 doses; 30 M cells	Day 1, 3 and 5.	Phase I, open-label, nonrandomized	Well-tolerated. No difference in the frequency of AE between the two groups.	*n* = 44 (26 MSC, 18 control) Significantly lowers the mortality of severe and critical SARS-CoV-2-induced COVID-19.	[115]
COVID-19 ARDS	UC-MSC	IV, 3 doses, 1 M cells/kg	Over 5 days after recruitment.	Phase 2b, RCT	Well-tolerated. No MSC- related AEs during treatment or thereafter (D28)	*n* = 45 (21 MSC, 24 control) No significant differences in oxygenation, PaO2/FiO2-Ratio change between D0-D7.	[116]
COVID-19 ARDS	BM-MSC	IV, 3 doses, 1.5–3 M cells/kg	3 (±1) day intervals	Phase I/II, open-label	Well tolerated. No MSC- related AEs	*n* = 32 (8 MSC, 24 control) MSC-treated had significantly lower day 7 D-dimer value compared to control patients.	[117]
COVID-19 ARDS	UC-MSC	IV, 2 doses, 100 M cells	Day 0 and 3	Phase I/IIa RCT	Well tolerated. No MSC- related AEs	*n* = 24 (12 MSC, 12 control) Inflammatory cytokines were significantly decreased in UC-MSC treated patients on day 6.	[94]
COVID-19 ARDS	UC-MSC	IV, 3 doses, 40 M cells	Day 0, 3 and 6	Phase 2 RCT	Well tolerated. Incidence of AE was similar in the two groups.	*n* = 100 (65 MSC, 35 control). UC-MSC groups exhibit an improvement in whole lung lesion volume from baseline to D28 compared to placebo.	[118]
COVID-19 ARDS	Placenta-derived Decidua Stromal Cells (DSCs)	IV,1–3 doses, 1 M cells/kg	During severe respiratory failure	Pilot study, open-label, Case series	Well tolerated. No severe infusion-related AEs.	*n* = 10 (10 DSC, no control group) DSC therapy exhibits a tendency of reduction of IL-6, G-CSF, CRP, CCL2 and, increase oxygenation and reversal of pulmonary disease. (Note: Results limited by pilot/open-label design biases)	[119]
COVID-19 ARDS	Placenta-derived Decidua Stromal Cells (DSCs)	IV, 1–2 doses, 1 M cells/kg		Pilot study, Open-label with propensity score-matched (PSM) controls	DSC administration appeared safe	*n* = 57 (19 DSC, 38 PSM control) Survival to hospital discharge was significantly higher for DSC-group compared to PSM control.	[120]
COVID-19 Pneumonia	UC-MSC	IV, single, 1 M cells/kg	Day 8	Phase I, RCT	Well-tolerated. No adverse events associated with MSC infusion	*n* = 40 (20 MSC, 20 Control). UC-MSC group showed a survival rate 2.5 times higher than control	[121]
Sepsis	Adipose MSCs	IV, 5 doses; 1 M cells/kg/dose	Soon after diagnosis of sepsis/shock	Phase I/II, non-randomized	Safe, no MSC-related AEs	Higher survival (days 7, 14, 28); decreased SOFA and APACHE II scores	[122]
Septic shock	BM-MSCs	IV, single; 0.3, 1.0, or 3.0 M cells/kg	24 h after ICU admission	Phase I, open-label dose escalation	Safe, well-tolerated at all doses	Stable biomarkers; efficacy conclusions precluded by Phase I design	[123]

AEs, adverse events; AKI, acute kidney injury; AR, adverse reactions; ARDS, acute respiratory distress syndrome; BM-MSC, bone marrow-derived mesenchymal stromal cell; C1/C2/C3, cohort 1/2/3; CPB, cardiopulmonary bypass; ECMO, extracorporeal membrane oxygenation; IV, intravenous; M, million; MAPC, multipotent adult progenitor cell; MB-MSC, menstrual-blood-derived mesenchymal stromal cell; PSM, propensity score-matched; RCT, randomized controlled trial; SP-D, surfactant protein D; UC-MSC, umbilical cord-derived mesenchymal stromal cell.

**Table 2:** Comparative Profile of Intact Mesenchymal Stromal Cells (MSCs) and MSC-Derived Extracellular Vesicles (EVs) for Clinical Translation.

Characteristic	Intact MSCs	MSC-Derived EVs
Physical size	15–50 µm	30–1000 nm (nanoscale)
Pulmonary first-pass effect	High; significant entrapment in the pulmonary capillary bed	Low; freely navigate the systemic microcirculation
Tumorigenicity risk	Potential risk due to capacity for self-replication (especially post-expansion)	None; lack a functional nucleus and replication machinery
Storage and stability	Requires toxic cryoprotectants (e.g., DMSO); susceptible to “cryo-impairment” on thawing	High stability at −80 °C; can be lyophilized; no DMSO required
Immunogenicity	Low, but can upregulate MHC-I/II under severe inflammatory stimulation	Extremely low; minimal surface expression of MHC antigens
Manufacturing bottlenecks	Limited by cellular senescence during large-scale in vitro expansion	Scalable, but requires complex, non-standardized isolation and purification techniques
Primary mechanism	Paracrine secretion, direct cell-to-cell contact, and mitochondrial transfer	Horizontal transfer of bioactive cargo (miRNAs, proteins, lipids, and organelles)

DMSO, dimethyl sulfoxide; MHC, major histocompatibility complex.

## Data Availability

No new data were created or analyzed in this study. Data sharing is not applicable to this article.

## Abbreviations

The following abbreviations are used in this manuscript:

AKI	Acute kidney injury
ARDS	Acute respiratory distress syndrome
AT	Adipose tissue
BM	Bone marrow
EV	Extracellular vesicles
GMP	Good manufacturing practice
IL	Interleukin
MODS	Multiple organ dysfunction syndrome
MSC	Mesenchymal stromal cell
NF	Nuclear factor
TEC	Tubular epithelial cells
TLR	Toll-like receptors
TNF	Tumor necrosis factor
UC	Umbilical cord
